# Minimising population health loss in times of scarce surgical capacity: a modelling study for surgical procedures performed in nonacademic hospitals

**DOI:** 10.1186/s12913-022-08854-x

**Published:** 2022-11-30

**Authors:** Anouk M. I. A. van Alphen, Kira S. van Hof, Benjamin Y. Gravesteijn, Eline M. Krijkamp, Pieter A. G. M. Bakx, Peter Langenbach, Jan J. Busschbach, Hester F. Lingsma, Robert J. Baatenburg de Jong, Value Based Operation Room Triage team collaborators, Arend Arends, Brigitte Haberkorn, Charles van Rossem, Gabrielle H. van Ramshorst, Han de Graaff, Harm Sleeboom, Jonne Postema, Josien Terwisscha van Scheltinga, Linda Valk-Kleibreuker, Marco Hoedt, Martin Baartmans, Mike Nieboer, Miriam Faes, Nieke Oversier, Niels Schep, Onno Schuitema, Patricia MacLean, Patrick Schouwenberg, Paul ten Koppel, Pieter Bakx, Sjaak Pouwels, Suze Raaff, Taco Nieboer, Tietse van Dorp, Willem Maarten Bosman

**Affiliations:** 1 Department of Otorhinolaryngology, Erasmus University Medical Center, Rotterdam, the Netherlands; 2 Department of Public Health, Erasmus University Medical Center, Rotterdam, the Netherlands; 3 Department of Epidemiology, Erasmus University Medical Center, Rotterdam, the Netherlands; 4 Currently Employed By the Erasmus School of Health Policy and Management, Erasmus University Rotterdam, Rotterdam, the Netherlands; 5 Department of Orthopedic Surgery, Maasstad Hospital, Rotterdam, the Netherlands; 6 CEO and Chairman of Maasstad Hospital, Rotterdam, the Netherlands; 7 Currently Employed By Zilveren Kruis (Achmea) Health Insurance, Leiden, the Netherlands; 8 Department of Medical Psychology, Erasmus University Medical Center, Rotterdam, the Netherlands

**Keywords:** Surgical planning, COVID-19, Decision modelling, Population health, Prioritisation, Quality of life

## Abstract

**Background:**

The burden of the COVID-19 pandemic resulted in a reduction of available health care capacity for regular care. To guide prioritisation of semielective surgery in times of scarcity, we previously developed a decision model to quantify the expected health loss due to delay of surgery, in an academic hospital setting. The aim of this study is to validate our decision model in a nonacademic setting and include additional elective surgical procedures.

**Methods:**

In this study, we used the previously published three-state cohort state-transition model, to evaluate the health effects of surgery postponement for 28 surgical procedures commonly performed in nonacademic hospitals. Scientific literature and national registries yielded nearly all input parameters, except for the quality of life (QoL) estimates which were obtained from experts using the Delphi method. Two expert panels, one from a single nonacademic hospital and one from different nonacademic hospitals in the Netherlands, were invited to estimate QoL weights. We compared estimated model results (disability adjusted life years (DALY)/month of surgical delay) based on the QoL estimates from the two panels by calculating the mean difference and the correlation between the ranks of the different surgical procedures. The eventual model was based on the combined QoL estimates from both panels.

**Results:**

Pacemaker implantation was associated with the most DALY/month of surgical delay (0.054 DALY/month, 95% CI: 0.025–0.103) and hemithyreoidectomy with the least DALY/month (0.006 DALY/month, 95% CI: 0.002–0.009). The overall mean difference of QoL estimates between the two panels was 0.005 (95% CI -0.014–0.004). The correlation between ranks was 0.983 (*p* < 0.001).

**Conclusions:**

Our study provides an overview of incurred health loss due to surgical delay for surgeries frequently performed in nonacademic hospitals. The quality of life estimates currently used in our model are robust and validate towards a different group of experts. These results enrich our earlier published results on academic surgeries and contribute to prioritising a more complete set of surgeries.

**Supplementary Information:**

The online version contains supplementary material available at 10.1186/s12913-022-08854-x.

## Background

Since the first identification of a novel coronavirus in December 2019, the rapid global spread of COVID-19 has challenged health care systems worldwide [1]. COVID-19 outcompeted regular care since critical care capacity and operation room (OR) resources were allocated to COVID-19 patients [2–4]. Consequently, the available surgical capacity was reduced and in the Netherlands this resulted in 75–90% fewer surgeries performed at the beginning of the COVID-19 crisis compared with previous years [4]. Likewise, the surgical capacity during the third COVID-19 wave (February until May 2021) was reduced by 21% [5]. The lack of optimal care for oncological, orthopaedic, ophthalmological, and cardiovascular patients resulted in higher mortality and morbidity rates, accompanied by a reduced quality of life [6–11]. Thus far, the “collateral damage” of COVID-19 continues to accumulate.

During the pandemic, health care experts have been facing dilemmas as to which patients to prioritise. In addition, the shortage of OR capacity has resulted in a significant backlog of elective or semielective surgeries [12, 13]. This gives rise to the debate on how to deal with the short- and long-term consequences of scarce surgical resources. As a multidisciplinary group of experts within the fields of psychology, ethics, medicine, and epidemiology, we developed a decision model to provide transparent and objective information to physicians in the triage process [14]. This model quantifies the consequences of delay for 43 surgeries in an academic setting. One of the input parameters used in this model is quality of life (QoL). Since these QoL weights were partly estimated by an expert panel and could therefore be disputed, a follow-up study was conducted with another expert panel to validate these QoL estimates [15]. The ranking of surgeries was remarkably unresponsive to which panel estimates were used.

Since our original decision model only simulated academic semielective surgical interventions, it does not apply to nonacademic hospital settings [14]. The aim of this study is to validate our decision model in a nonacademic setting and include additional elective surgical procedures. This extension is especially of added value since the current prioritisation dilemmas mostly involve this type of surgery.

## Methods

### Markov model

We used a previously developed decision model to assess the health effects of surgery postponement of surgeries frequently performed in nonacademic hospitals. This model is a three-state cohort state-transition model, often called a Markov model, and was originally used to assess the health effects of surgery postponement in the academic setting. A detailed model description can be found in our previously published work [14].

The three health states considered in this model were the preoperative, postoperative, and dead states (see Additional file 1). The entire cohort started in the preoperative state, followed by a transition to the postoperative or dead state. The scenarios of surgical delay were modelled with intervals of ten weeks, starting from two weeks up to one year. Definitive cancellation of surgery was modelled as patients remaining in the preoperative health state for their remaining lifetime until they became 100 years old. The model required seven input parameters: 1) survival rate presurgery, 2) survival rate postsurgery, 3) QoL presurgery, 4) QoL postsurgery, 5) mean age of patients undergoing the surgery, 6) time until no effect of treatment can be expected on survival or 7) time until no effect of treatment can be expected on QoL (see Additional file 1). The model outcomes were years of life lost (YLL) and disability adjusted life years (DALY), which is similar to the outcome used in our study on surgeries performed in the academic setting. A detailed description of the methods used to calculate model outcome can be found in our previous work [14]. DALYs were preferred as the leading urgency measure since they incorporate QoL in contrast to YLL, and disability weights were used to calculate health loss. With respect to the utilitarian ethical perspective, priority should be given to patients with the most DALYs per unit of time since this approach will minimise total health loss for society. Health outcomes were calculated based on the expected outcome at 52 weeks compared with the outcome at two weeks of surgical delay. The difference yields the health loss per 50 weeks. This measure is converted to health loss per month delay. This urgency measure yields the ranking of the surgical procedures, where procedures with a high DALY/month are ranked higher.

### Surgery selection

A list of the 100 most frequently performed surgeries in nonacademic hospitals in the Netherlands in 2019 was obtained from the Dutch Hospital Data (DHD) national registry [16]. Subsequently, acute, paediatric, and obstetric surgeries were excluded by a senior physician (RBdJ). Acute was defined as interventions that had to be performed within 72 h after hospital admission. Surgeries performed at the outpatient clinic were also excluded, assuming the outpatient clinic capacity was less affected by COVID-19 compared with the reduction in operating room capacity.

### Literature data

The input parameters mean age and survival pre- and postsurgery were obtained from the National Heart Registry (NHR), Dutch Hospital Data, and Netherlands Cancer Registry (NCR) [16–18]. Registry data were complemented with input from the scientific literature. This extensive literature search also generated relevant data on the two input parameters of ‘time until no effect’ on survival and QoL. In cases of insufficient or missing data, physicians specialized in this specific surgery were consulted to give their considered opinion. All input parameters were classified as class I (Randomized Controlled Trials (RCT) or systematic reviews of RCTs), class IIa (Prospective observational studies, before-after studies), class IIb (Retrospective observational studies, expert panels for utilities, national registries), and class III (expert opinion).

### Quality of life collection

To estimate the QoL input parameters, a two-round Delphi study was conducted. The preparation phase consisted of participant recruitment and establishing the method for QoL collection. Medical experts from surgical and nonsurgical specialties were asked to participate in an online panel (Value Based Operation Room Triage team collaborators, see Additional file 3). The primary expert panel consisted of 15 medical experts from a general teaching hospital affiliated with our academic centre (Maasstad Hospital Rotterdam), named ‘Maasstad experts’. Through snowball sampling, another 11 medical experts from different nonacademic hospitals in the Netherlands were recruited, called ‘Other experts’. They were asked to participate in a comparable Delphi study to validate the results of the primary expert panel.

QoL data were collected in accordance with the methods described by Stouthard et al., whereby health states are rated according to a given scale [19]. First, case descriptions were established for every surgery (*n* = 28) and illustrated a preoperative and postoperative health state. These descriptions were established after consultation with an external senior physician. For procedures in accordance with our previous study on academic surgeries, the descriptions were adjusted to the nonacademic setting. For both expert panels, the first round of the Delphi study started in August 2020. Due to COVID-19 restrictions, we used an online web-based Delphi method tool named Welphi [20]. The medical experts were asked to rate the QoL for each health state on a calibrated visual analogue scale (VAS). This scale ranged from 0 (being the worst health state imaginable, similar to death) to 100 (being the best health state imaginable). Available QoL estimates from the WHO Global Burden of Disease were presented to the participants as reference points (see Additional file 3) [21].

Additionally, they were asked to write a short commentary with their arguments for the given scores. These comments, accompanied by the median score and interquartile range of the entire expert group, were displayed during the second round in October 2020. Each individual was able to adjust his or her QoL valuations based on this additional information, approaching group consensus. A detailed overview of the QoL collection method is presented in our earlier publications [14, 15].

### Analysis

The published decision model was used for our analysis. This decision model was developed in the open-source software R [22] and based on coding recommendations for decision analysis in health care [23–25]. The full model code is available on GitHub via the following link: https://github.com/bgravesteijn/ Utilitarian-distribution-of-OR-capacity-during-COVID-19. The decision model includes a probabilistic sensitivity analysis that is used to reflect parameter uncertainty in the model results [26].

A Bland–Altman analysis was performed to analyse the agreement between the QoL estimates from the ‘Maasstad experts’ and the ‘Other experts’ [27, 28]. The pre- and postoperative QoL estimates were analysed separately. Lower and upper levels of agreement were calculated. Furthermore, the overall mean difference in QoL estimates between the expert panels was calculated by fitting a linear mixed effects model with a random intercept for surgical procedures. Thereafter, the effect of using other QoL estimates on the DALY/month estimates and ultimate ranking of surgical procedures was evaluated using Spearman’s rank correlation; the QoL estimates from the ‘Maasstad experts’ were used as input parameters in the decision model, which yielded the original ranking of surgical procedures and DALY/month. Then, the new QoL estimates retrieved from the other expert panel were utilised as input. These new DALY/month estimates accompanied by the corresponding ranking, were compared with the results of the primary data only. Analyses were conducted using R software [22]. The lmer function in the lme4 package was used to fit the linear mixed effects model [29].

## Results

**Fig. 1:**
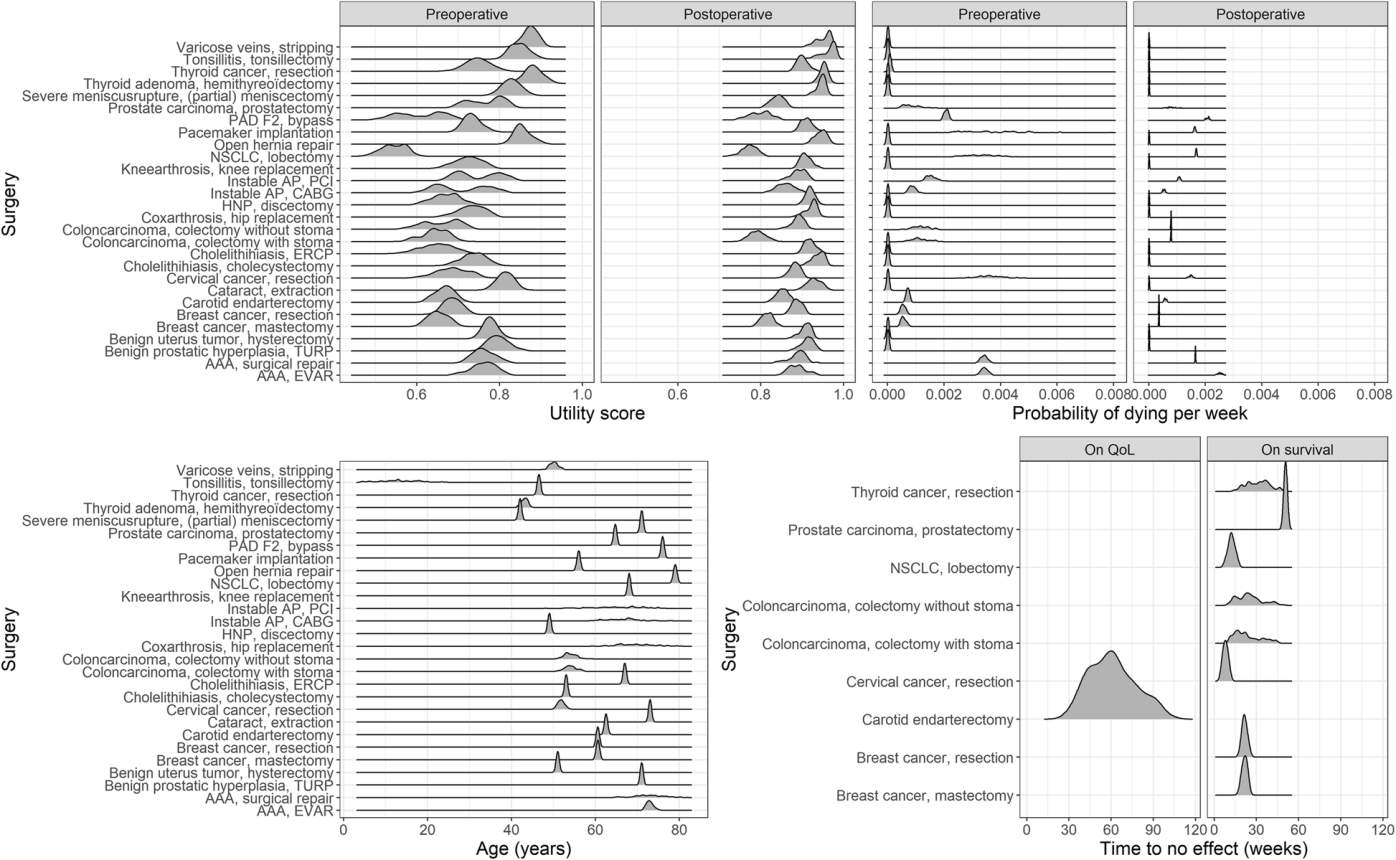
Final distribution of the seven input parameters used during probabilistic sensitivity analysis (PSA). y-axis = surgeries; x-axis = parameters. See Table 3 for the abbreviations used

In total, 28 surgeries were selected and evaluated with the decision model. This selection comprised procedures from the following surgical specialties: general (*n* = 7), cardiothoracic (*n* = 5), vascular (*n* = 5), orthopaedic (*n* = 3), gynaecological (*n* = 2), urological (*n* = 2), ear, nose and throat (*n* = 1), endocrine (*n* = 1), neurosurgical (*n* = 1), and ophthalmological (*n* = 1) (see Additional file 2, Table A2.1). Both pre- and postoperative survival were mostly based on class III evidence (13 out of 28, Table 1). For eight surgical procedures, survival was preoperatively calculated through the treatment effect found in the literature (class IIb evidence). Time to no effect on QoL was based on class I evidence. Time to no effect on survival was assumed for eight surgical procedures, all based on class II evidence. For all 28 procedures, the QoL weights were estimated by the expert panels (see Table 1). All parameters were varied in sensitivity analyses (Fig. 1).

**Table 1:** List of frequently used abbreviations in the manuscript

Abbreviation	Meaning
AAA	Aneurysm of the abdominal aorta
AP	Angina pectoris
ASD	Atrial septum defect
AVR	Aortic valve replacement
CABG	Coronary artery bypass graft
COPD	Chronic obstructive pulmonary disease
COVID-19	Coronavirus disease 2019
DALY	Disability adjusted life years
DHD	Dutch Hospital Data
ENT	Ear, Nose and Throat
ERCP	Endoscopic retrograde cholangiopancreatography
ESHF	End-stage heart failure
ESLD	End-stage liver disease
ESRD	End-stage renal disease
EVAR	Endovascular aortic repair
HCC	Hepatocellular cancer
HIPEC	Hyper thermic intraperitoneal chemotherapy
HNP	Herniated nucleus pulposus
ICD	Implantable cardioverter-defibrillator
ICU	Intensive care unit
LVAD	Left ventricle assist device
MI	Muscle invasive
MVP	Mitral valve plasty
NA	Nonacademic
NCR	Netherlands Cancer Registry
NHR	National Heart Registry
NSCLC	Non-small cell lung cancer
OR	Operating Room
PAD F2	Peripheral arterial disease Fontaine classification 2
PAD F3-4	Peripheral arterial disease Fontaine classification 3–4
PCI	Percutaneous coronary intervention
Postop	Postoperative
Preop	Preoperative
PSA	Probabilistic Sensitivity Analysis
QoL	Quality of life
RCT	Randomized Controlled Trial
St	Stage
Symp +	Symptomatic
TAVI	Transaortic valve implantation
Time no eff	Time until no effect on quality of life/survival
TURP	Transurethral resection of the prostate
UUT	Upper urinary track
VAS	Visual analogue scale
VATS	Video-assisted thoracoscopic surgery
WHO	World Health Organization
YLL	Years of life lost

### QoL estimates

**Fig. 2:**
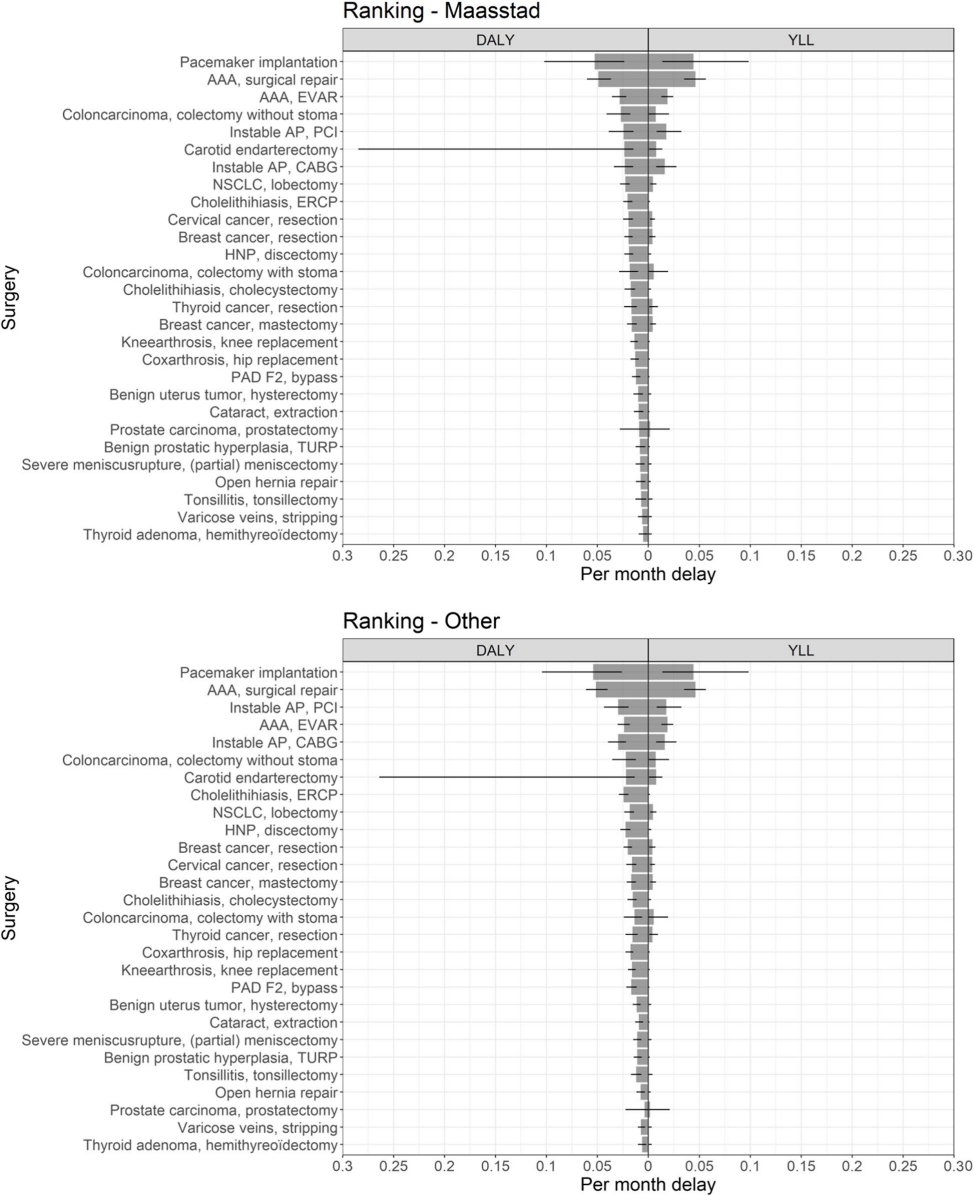
Ranking established using QoL estimates from ‘Maasstad experts’ (top) and ‘Other experts’ (below). The average DALYs and YLLs per month of surgical delay are displayed for the surgeries frequently performed in nonacademic hospitals. The estimates (gray bars) and 95% confidence intervals (black lines) are shown. The 95% confidence interval for ‘carotid endarterectomy’ can be explained by the high uncertainty in the ‘time to no effect’ parameter. See Table 3 for the abbreviations used

Fifteen physicians from Maasstad Hospital participated, 6 of whom were from surgical specialities. The panel of 11 ‘Other experts’ that validated the primary QoL estimates had approximately the same ratio of surgical to nonsurgical specialities as the 15 ‘Maasstad experts’ (see Table 2). The QoL weights estimated by the two panels were very similar (see Fig. 2). The overall mean difference between these two panels was 0.005 (95% CI -0.014–0.004). The Bland–Altman mean bias and lower and upper levels of agreement were -0.009 (-0.101–0.083) for the preoperative estimates, and -0.001 (-0.033–0.031) for the postoperative estimates (see Additional file 4).

**Table 2:** Characteristics of the type and class of evidence incorporated into the decision model

	Age	QoL – Preop	QoL – Postop	Survival – Preop	Survival – Postop	Time no eff – Survival	Time no eff – QoL	Treatment effect
n	28	28	28	28	28	8	1	8
**Type of evidence (%)**								
Before-after study	0 (0)	0 (0)	0 (0)	0 (0)	0 (0)	0 (0)	0 (0)	0 (0)
Expert opinion	0 (0)	0 (0)	0 (0)	13 (46.4)	13 (46.4)	0 (0)	0 (0)	1 (12.5)
Expert panel	0 (0)	28 (100)	28 (100)	0 (0)	0 (0)	0 (0)	0 (0)	0 (0)
Expert panel (WHO)	0 (0)	0 (0)	0 (0)	0 (0)	0 (0)	0 (0)	0 (0)	0 (0)
National registry	16 (57.1)	0 (0)	0 (0)	2 (7.1)	10 (35.7)	2 (25.0)	0 (0)	1 (12.5)
Observational, prospective	1 (3.6)	0 (0)	0 (0)	0 (0)	1 (3.6)	4 (50.0)	0 (0)	0 (0)
Observational, retrospective	6 (21.4)	0 (0)	0 (0)	6 (21.4)	3 (10.7)	2 (25.0)	0 (0)	1 (12.5)
RCT	5 (17.9)	0 (0)	0 (0)	7 (25.0)	1 (3.6)	0 (0)	1 (100)	5 (62.5)
**Class of evidence (%)**								
I	5 (17.9)	0 (0)	0 (0)	7 (25.0)	1 (3.6)	0 (0)	1 (100)	5 (62.5)
IIa	1 (3.6)	0 (0)	0 (0)	0 (0)	1 (3.6)	4 (50.0)	0 (0)	0 (0)
IIb	22 (78.6)	28 (100)	28 (100)	8 (28.6)	13 (46.4)	4 (50.0)	0 (0)	2 (25.0)
III	0 (0)	0 (0)	0 (0)	13 (46.4)	13 (46.4)	0 (0)	0 (0)	1 (12.5)

Class definitions: I = RCT or systematic reviews of RCTs; IIa = prospective observational studies, before-after studies, *IIb* retrospective observational studies, expert panels for the utilities, national registries; class III = expert opinion. *QoL* quality of life, *WHO* World Health Organization

**Table 3:** Characteristics of the two expert panels which estimated the QoL weights

Speciality	Maasstad experts	Other experts
Surgical speciality		
ENT surgeon	1	
General surgeon	1	3
Oncological gynaecologist	1	
Orthopaedic surgeon	1	1
Trauma surgeon	1	
Plastic surgeon	1	
	6 (40% of total)	4 (36% of total)
Non-surgical speciality		
Anaesthesiologist	4	
General practitioner		2
Geriatrician	1	2
Internist	1	1
Paediatrician	1	
Radiologist		1
Rehabilitation physician	1	
Resident not in training (ICU)		1
Rheumatologist	1	
Total	15	11

The ‘Maasstad experts’ make up the panel of the affiliated hospital. The panel of ‘Other experts’ was composed to validate these estimates. See Table 3 from the main manuscript for the abbreviations used

### Ranking

**Fig. 3:**
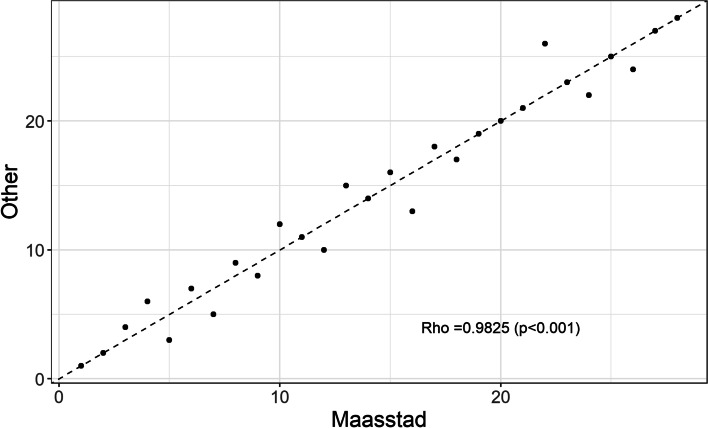
Correlation between rankings based on QoL estimates from ‘Maasstad experts’ (x-axis) versus ‘Other experts’ (y-axis). Rho = Spearman correlation coefficient

Since the QoL estimates were comparable between the two panels, the definitive ranking of surgical procedures was relatively unresponsive to the estimates used. Spearman’s correlation coefficient between the primary ranking and the ranking based on QoL estimates of the ‘Other experts’ was rho = 0.983 (*p* < 0.001) (see Fig. 3). The largest difference in DALY/month was found for ‘instable AP, CABG’. Using the original QoL estimates from ‘Maasstad experts’, the DALY/month was 0.023 (95% CI 0.015–0.034), whereas using the QoL estimates from the ‘Other experts’ yielded 0.030 (95% CI 0.022–0.040) DALY/month. This decrease in DALY/month resulted in a shift from place seven to place five (see Fig. 2).

**Fig. 4:**
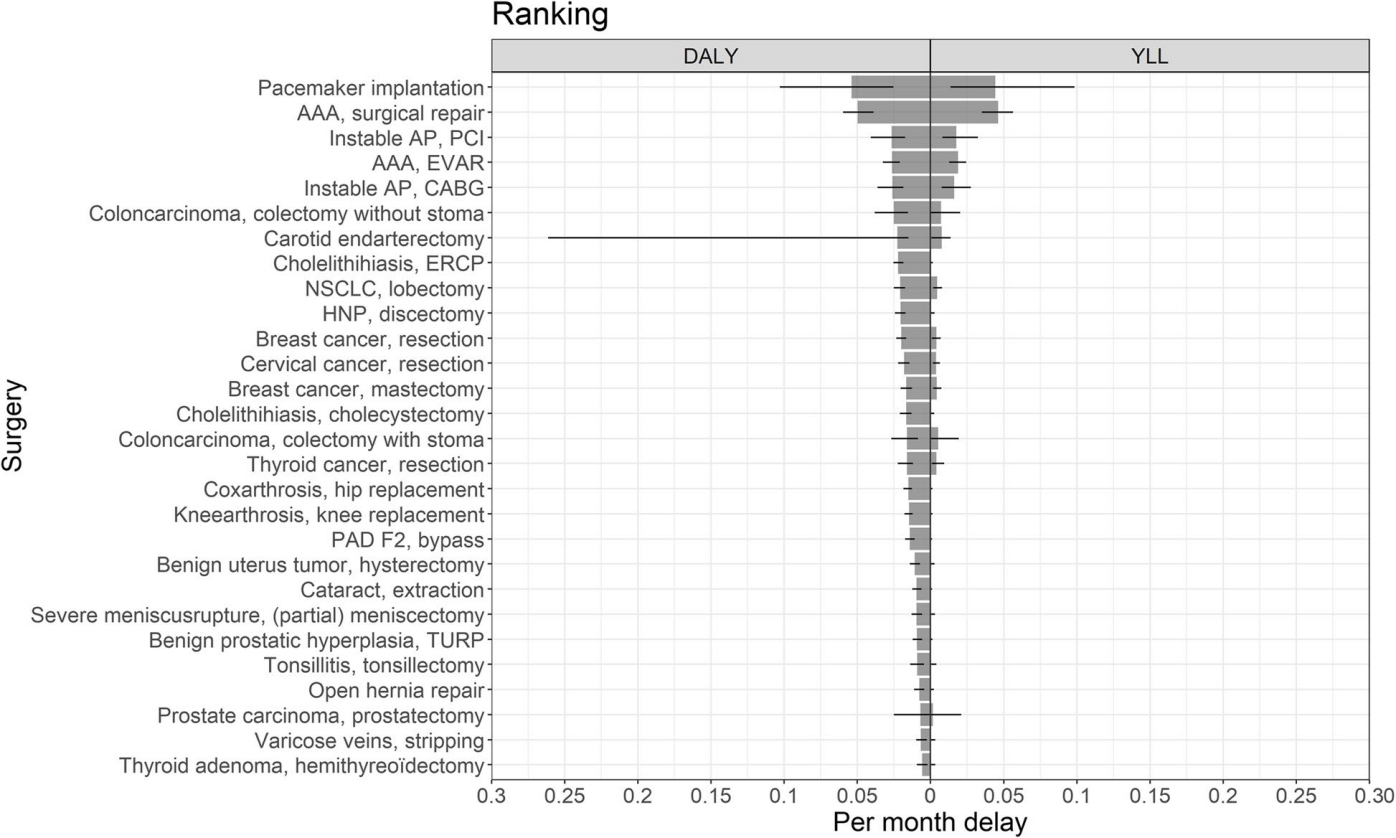
Final ranking of n = 28 surgeries frequently performed in nonacademic hospitals. This ranking incorporates QoL estimates of both expert panels. The average DALYs and YLLs per month of surgical delay are displayed. The estimates (gray bars) and 95% confidence intervals (black lines) are shown. See Table 3 for the abbreviations used

The DALYs resulting from one month of surgical delay ranged from 0.006 (95% CI 0.002–0.009) for performing a hemithyreoidectomy in patients with thyroid adenoma to 0.054 (95% CI 0.025–0.103) for pacemaker implantation (see Fig. 4 for an overview of all 28 surgeries). This ranking was established using the QoL estimates of both expert panels and considered the definitive ranking.

**Fig. 5:**
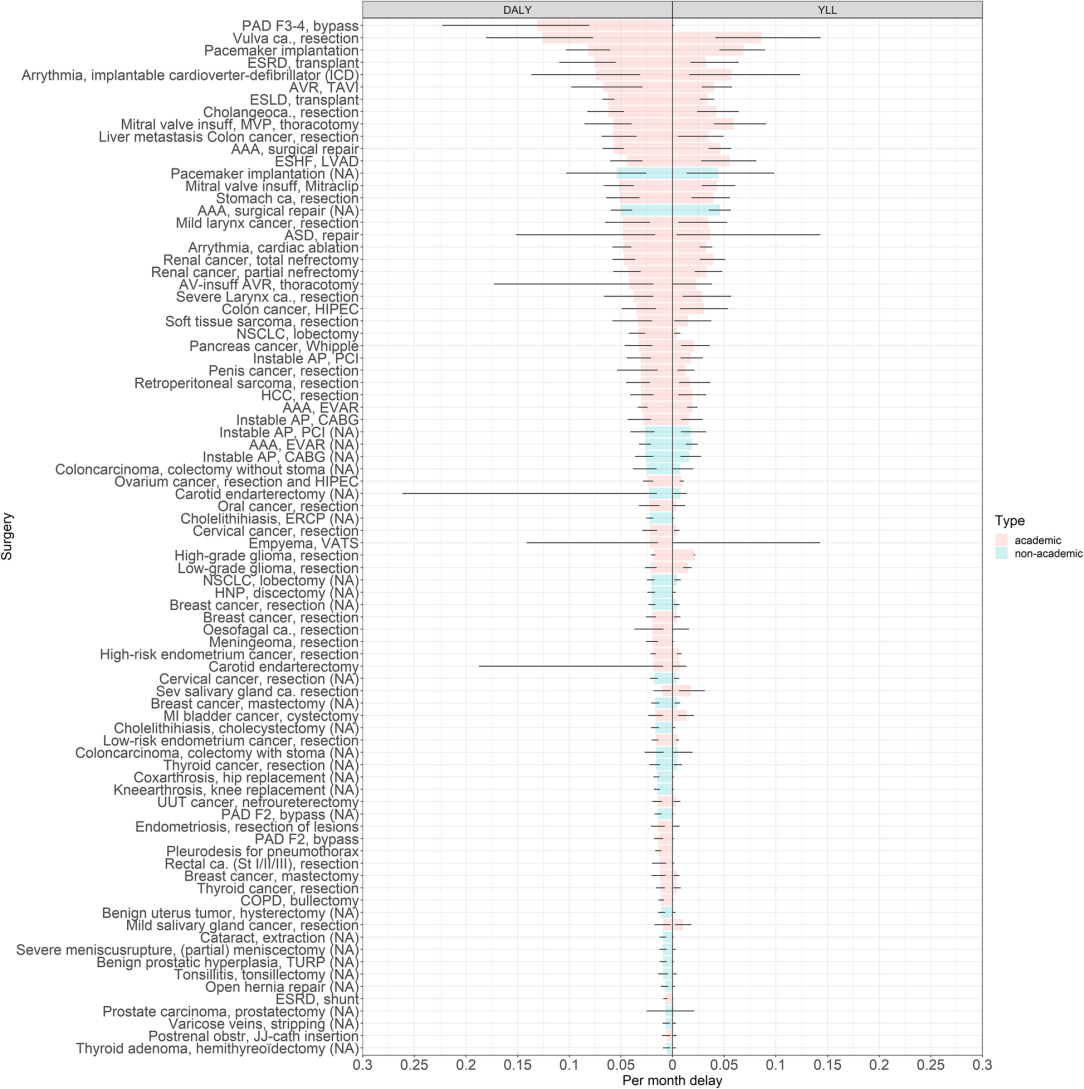
Ranking of *n* = 84 surgeries (performed in nonacademic and academic hospitals) incorporated into our model. The average DALYs and YLLs per month of surgical delay are displayed. The estimates (colored bars) and 95% confidence intervals (black lines) are shown. The red bars represent academic surgeries and the blue bars represent nonacademic surgeries. NA = nonacademic. See Table 3 for the abbreviations used

Eventually, all currently available (*n* = 84) were integrated into an online tool where users can select different surgeries and compare the surgical outcome applicable to their hospital setting. This tool is accessible via OR triage decision tool (shinyapps.io). Figure 5 presents an overview of all surgeries currently incorporated in our model. As shown in this figure, the ranking based on YLL/month, which incorporates survival only, is comparable to the ranking of DALY/month, where QoL is also taken into account.

## Discussion

In this study, we estimated the health consequences of surgical delay for 28 surgeries frequently performed in a nonacademic setting. This is an extension of our previously published work and contributes to a better overview of the entire mix of surgeries performed in both academic and nonacademic hospitals. The surgical intervention associated with the most DALYs/month due to surgical delay, was a pacemaker implantation. Hemithyreoidectomy was the procedure with the least health loss per month delay. Furthermore, we showed that the QoL estimates used as input parameters for the model are robust and validate towards different groups of experts.

The results of this study further bolster our previous findings on surgeries performed in academic hospitals. By extending the model to a nonacademic setting, we included 16 new procedures and elective surgical interventions that mostly intended to avert QoL loss. This extension is especially of added value for clinical practice since the current worldwide backlog of surgeries mainly consists of elective procedures [5, 30–32]. Moreover, the current differentiation in hospital setting enables health care professionals to obtain an estimation of the expected health loss, which is applicable to their hospital setting. Whenever the model outcome does not correspond to their setting, it is crucial to take the patient’s health status into consideration. For fragile and comorbid patients, it could be argued that the likelihood that they will benefit from surgery is lower than that for relatively healthy patients [33]. This argument, however, is inherently related to the controversy over using utilities to allocate resources in general [34].

Our analysis showed that the ranking of surgeries frequently performed in nonacademic hospitals is primarily driven by survival and not QoL. The ranking based on YLL/month was comparable to the definitive ranking of DALY/month. This accords with our earlier results describing semielective surgeries [14]. In the current study, we added elective procedures, which are nonemergency surgeries for conditions that are by definition not an immediate threat to life [35, 36]. Since the impact of postponing these elective surgeries on YLL is relatively low compared with semielective surgeries, nonemergency surgical interventions (i.e., tonsillectomy) ended up lower in the ranking. However, it should be noted that the delay of these surgeries could still lead to unintended physical consequences, worsened quality of life and subsequently societal costs through decreased work productivity for patients [11, 37–39].

Other studies have emphasised the resource allocation dilemmas that occurred during the COVID-19 pandemic and subsequently suggested several strategies to counter them [40–43]. These strategies arise from different ethical principles that could all provide guidance but come with different practical implications. Our decision model is consistent with the utilitarian perspective: striving for the greatest good for the greatest number of people. The decision for this ethical principle was made deliberately, since utilitarianism was most preferred by health care professionals in the context of a pandemic [41, 44–46].

In the literature, the agreement on prioritisation between surgeons is low, especially between different specialties [47, 48]. A major advantage of our model is that it objectifies the consequences of prioritisation decisions, which otherwise remain implicit. This property helps to structure the discussion on prioritisation between health care professionals of different specialities and policymakers, as they are more objectively informed. With this model, we integrate the best available scientific evidence and merge this with validated expert opinions. The model outcome (DALY/month delay) enables direct comparisons of surgeries by providing a common outcome measure to quantify the impact of surgery postponement across disciplines. The current extension facilitates intraregional arrangements on prioritisation as a mix of surgeries from different settings is modelled.

The issues of prioritisation and rationing are both ethically and politically sensitive. The idea of prioritising surgical patients based on our decision model might lead to a feeling of discomfort for physicians. The urgency ranking should therefore be considered as an additional information tool to make a deliberate and concise decision. On the whole, the model outcome could be of great value to health care professionals worldwide during the COVID-19 pandemic. The model input could be adjusted to country or region-specific data, which makes the output applicable to a specific setting. Furthermore, we also believe that value-driven resource allocation will be useful after the pandemic. Other causes such as budgetary constraints or personnel shortages, will ultimately lead to scarce resources, thereby forcing physicians once more to prioritise patients.

## Strengths and limitations

The limitations of the decision model we used have previously been published and are again applicable to the current study [14]. In summary, we made several assumptions in our model, thereby simplifying the complex process of prioritising surgical patients and disregarding the potential risks of surgery itself. Moreover, the validity and generalisability of the model outcome are highly dependent on the quality of the input parameters, which might have affected the results. In particular, the expected survival without surgery comes with substantial uncertainty. For the current study, it could be argued that the QoL estimates obtained from 26 physicians might not generally be applicable to all patients diagnosed with the included diseases. To conclude, a major limitation to using the model results in practice remains the limited number of surgeries that were modelled.

This study strengthens the usefulness of our previously published work by describing surgeries in the nonacademic setting. Further research should focus on incorporating patient characteristics that are known to influence perioperative or postoperative surgical outcomes, e.g., age and comorbidities. By this means, the model outcome can become much more refined, thereby further approaching the real-life clinical situation. Furthermore, major stakeholders (e.g., physicians, patients) are approached to evaluate any ethical or contextual considerations that should be considered for successful implementation of our model. Moreover, metrics from capacity management (OR time, complications, length of stay) are currently fed into the model. This could make the model helpful for capacity management, and it will provide insights into what combination of surgeries would yield the least health loss given a certain capacity constraint. Last, it is of utmost importance that the model’s validity is preserved by integrating the best available evidence and ensuring that it remains up to date.

## Conclusion

We extended our model to surgical procedures in a nonacademic setting and validated the QoL estimates used in that extension. This extended model now incorporates 84 (semi)elective surgeries, including surgeries frequently performed in academic and nonacademic hospitals. We provide an objective framework as a starting point in the discussion on how to prioritise surgical patients, acknowledging the methodological and ethical assumptions made. Our model could help to optimise the utilisation of available surgical capacity in a transparent way in times of scarce surgical capacity and/or when countries are dealing with an extensive backlog of (semi)elective surgeries.

## Supplementary Information

**Additional file 1.** 

**Additional file 2.** 

**Additional file 3.** 

**Additional file 4.**

## Data Availability

All data generated or analysed during this study are included in this published article and its supplementary information files.
