# Supplementary material for: Minimising population health loss in times of scarce surgical capacity: a modelling study for surgical procedures performed in nonacademic hospitals
Source: BMC Health Serv Res. 2022 Nov 30;22:1456. doi: 10.1186/s12913-022-08854-x (PMC9713162; doi:10.1186/s12913-022-08854-x)
Supplement: Supplementary file 1 — Additional file 1. [file 12913_2022_8854_MOESM1_ESM.docx]

Additional file 1

An overview of the model structure and the input parameters.

A three-state cohort state-transition model was previously developed and used to simulate the effect on health loss due to surgical delay (14). Preop: preoperative, postop: postoperative.


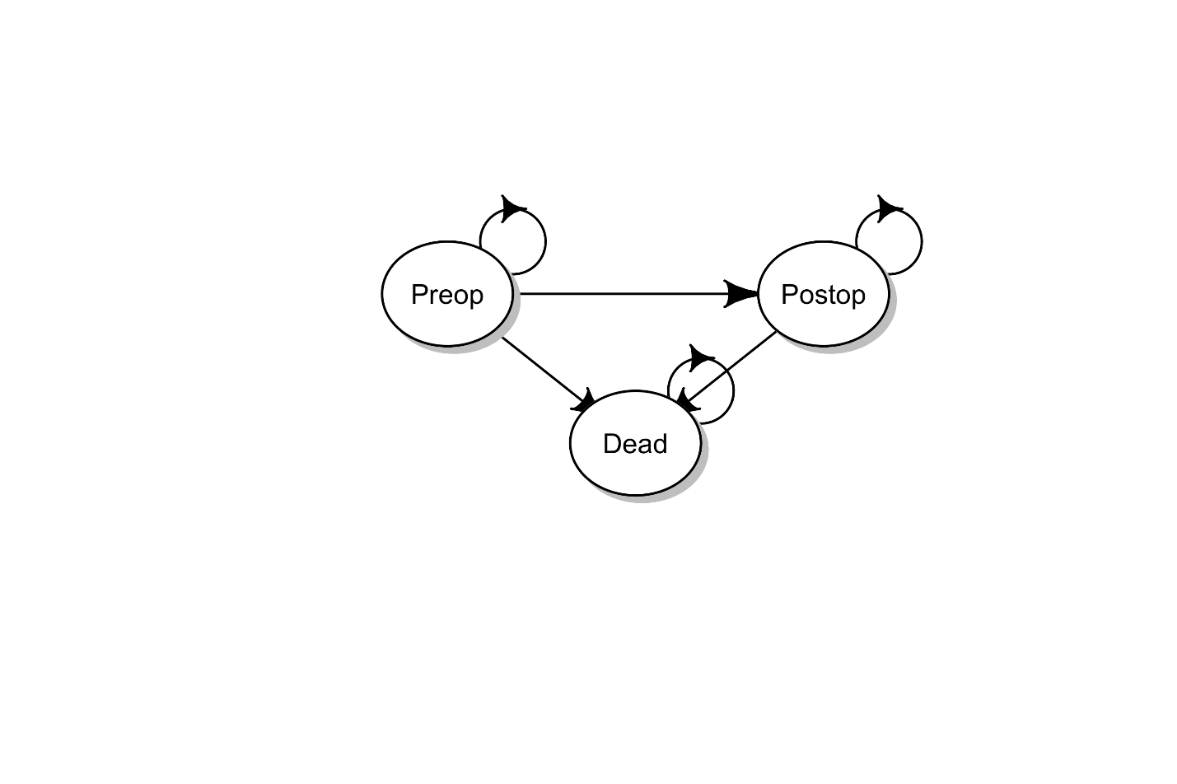


Input parameters of the model.

| **Parameter** | **Description** |
| --- | --- |
| Age | Average age of the patient population. |
| Preoperative survival rate | The survival rate of patients before the surgical procedure. |
| Postoperative survival rate | The survival rate of patients after the surgical procedure. |
| Preoperative quality of life | The quality of life of patients before the surgical procedure. |
| Postoperative quality of life | The quality of life of patients after the surgical procedure. |
| Time until no effect on quality of life | The time until no effect from the procedure can be expected on quality of life. |
| Time until no effect on survival | The time until no effect from the procedure can be expected on survival. |
