# Supplementary material for: Minimising population health loss in times of scarce surgical capacity: a modelling study for surgical procedures performed in nonacademic hospitals
Source: BMC Health Serv Res. 2022 Nov 30;22:1456. doi: 10.1186/s12913-022-08854-x (PMC9713162; doi:10.1186/s12913-022-08854-x)
Supplement: Supplementary file 2 — Additional file 2. [file 12913_2022_8854_MOESM2_ESM.docx]

Additional file 2

A summary of the nonacademic surgeries incorporated into our model and the model outcome. Per surgery, the distribution and source of the input parameters is presented.

Table A2.1. Total of 28 surgical procedures frequently performed in a nonacademic setting which were incorporated into our model and their associated model outcome (DALY/month), ranked from high to low DALY/month.

|  | Surgery | DALY/month |
| --- | --- | --- |
| 1 | Pacemaker implantation | 0.054 (0.025 - 0.103) |
| 2 | AAA, surgical repair | 0.050 (0.039 - 0.060) |
| 3 | Instable AP, PCI | 0.027 (0.017 - 0.041) |
| 4 | AAA, EVAR | 0.026 (0.021 - 0.033) |
| 5 | Instable AP, CABG | 0.026 (0.018 - 0.036) |
| 6 | Colon carcinoma, colectomy without stoma | 0.025 (0.015 - 0.038) |
| 7 | Carotid endarterectomy | 0.023 (0.015 - 0.261) |
| 8 | Cholelithihiasis, ERCP | 0.022 (0.019 - 0.025) |
| 9 | NSCLC, lobectomy | 0.021 (0.017 - 0.025) |
| 10 | HNP, discectomy | 0.020 (0.017 - 0.024) |
| 11 | Breast cancer, resection | 0.020 (0.017 - 0.023) |
| 12 | Cervical cancer, resection | 0.018 (0.014 - 0.022) |
| 13 | Breast cancer, mastectomy | 0.017 (0.013 - 0.021) |
| 14 | Cholelithihiasis, cholecystectomy | 0.017 (0.013 - 0.021) |
| 15 | Colon carcinoma, colectomy with stoma | 0.016 (0.009 - 0.027) |
| 16 | Thyroid cancer, resection | 0.016 (0.012 - 0.022) |
| 17 | Cox arthrosis, hip replacement | 0.015 (0.013 - 0.019) |
| 18 | Knee arthrosis, knee replacement | 0.015 (0.012 - 0.018) |
| 19 | PAD F2, bypass | 0.014 (0.011 - 0.017) |
| 20 | Benign uterus tumor, hysterectomy | 0.011 (0.007 - 0.014) |
| 21 | Cataract, extraction | 0.010 (0.006 - 0.013) |
| 22 | Severe meniscusrupture, (partial) meniscectomy | 0.010 (0.006 - 0.013) |
| 23 | Benign prostatic hyperplasia, TURP | 0.009 (0.006 - 0.012) |
| 24 | Tonsillitis, tonsillectomy | 0.009 (0.005 - 0.014) |
| 25 | Open hernia repair | 0.008 (0.004 - 0.011) |
| 26 | Prostate carcinoma, prostatectomy | 0.007 (0.006 - 0.025) |
| 27 | Varicose veins, stripping | 0.007 (0.003 - 0.010) |
| 28 | Thyroid adenoma, hemithyreoïdectomy | 0.006 (0.002 - 0.009) |

Abbreviations: AAA, aneurysm of the abdominal aorta; AP, angina pectoris; CABG, coronary artery bypass graft; ERCP, endoscopic retrograde cholangiopancreatography; EVAR, endovascular aortic repair; HNP, herniated nucleus pulposus, NSCLC, non-small cell lung cancer; PAD F2, peripheral arterial disease Fontaine classification 2; PCI, percutaneous coronary intervention; TURP, transurethral resection of the prostate.

Table 2. An overview per surgery of the distribution and source of the input parameters.

| **Surgery** | **Parameter** | **Source** | **PSA estimate (95% CI)** | **Distribution** |
| --- | --- | --- | --- | --- |
| Cataract, extraction | Surv_no_tx | Clinical insight | 1.00 (1.00 - 1.00) | Triangle |
| Cataract, extraction | Surv_tx | Clinical insight | 1.00 (1.00 - 1.00) | Triangle |
| Cataract, extraction | QoL_no_tx | Experts | 0.81 (0.79 - 0.84) | Triangle |
| Cataract, extraction | QoL_tx | Experts | 0.93 (0.91 - 0.95) | Triangle |
| Cataract, extraction | Age | DHD | 73.00 (72.95 - 73.04) | Normal |
| Open hernia repair | Surv_no_tx | Clinical insight | 1.00 (1.00 - 1.00) | Triangle |
| Open hernia repair | Surv_tx | Clinical insight | 1.00 (1.00 - 1.00) | Triangle |
| Open hernia repair | QoL_no_tx | Experts | 0.85 (0.83 - 0.88) | Triangle |
| Open hernia repair | QoL_tx | Experts | 0.95 (0.93 - 0.96) | Triangle |
| Open hernia repair | Age | DHD | 55.95 (55.57 - 56.41) | Normal |
| Cholelithihiasis, cholecystectomy | Surv_no_tx | Clinical insight | 1.00 (1.00 - 1.00) | Triangle |
| Cholelithihiasis, cholecystectomy | Surv_tx | Clinical insight | 1.00 (1.00 - 1.00) | Triangle |
| Cholelithihiasis, cholecystectomy | QoL_no_tx | Experts | 0.73 (0.70 - 0.77) | Triangle |
| Cholelithihiasis, cholecystectomy | QoL_tx | Experts | 0.94 (0.92 - 0.95) | Triangle |
| Cholelithihiasis, cholecystectomy | Age | DHD | 52.99 (52.81 - 53.16) | Normal |
| Cholelithihiasis, ERCP | Surv_no_tx | Clinical insight | 1.00 (1.00 - 1.00) | Triangle |
| Cholelithihiasis, ERCP | Surv_tx | Clinical insight | 1.00 (1.00 - 1.00) | Triangle |
| Cholelithihiasis, ERCP | QoL_no_tx | Experts | 0.65 (0.61 - 0.69) | Triangle |
| Cholelithihiasis, ERCP | QoL_tx | Experts | 0.92 (0.90 - 0.94) | Triangle |
| Cholelithihiasis, ERCP | Age | DHD | 66.96 (66.68 - 67.27) | Normal |
| Tonsillitis, tonsillectomy | Surv_no_tx | Clinical insight | 1.00 (1.00 - 1.00) | Triangle |
| Tonsillitis, tonsillectomy | Surv_tx | Clinical insight | 1.00 (1.00 - 1.00) | Triangle |
| Tonsillitis, tonsillectomy | QoL_no_tx | Experts | 0.85 (0.83 - 0.87) | Triangle |
| Tonsillitis, tonsillectomy | QoL_tx | Experts | 0.96 (0.94 - 0.98) | Triangle |
| Tonsillitis, tonsillectomy | Age | DHD | 12.98 (4.53 - 23.17) | Normal |
| AAA, surgical repair | Surv_no_tx | Scott S, Batchelder A, Kirkbride D, et al. Eur J Vasc Endovasc Surg 2016;52(4):444-449 | 0.84 (0.83 - 0.85) | Triangle |
| AAA, surgical repair | Surv_tx | Nathan D, Brinster C, Jackson B, et al. J Vasc Surg 2011;54(5)1237-43 | 0.92 (0.92 - 0.92) | Triangle |
| AAA, surgical repair | QoL_no_tx | Experts | 0.76 (0.73 - 0.80) | Triangle |
| AAA, surgical repair | QoL_tx | Experts | 0.89 (0.86 - 0.92) | Triangle |
| AAA, surgical repair | Age | Nathan D, Brinster C, Jackson B, et al. J Vasc Surg 2011;54(5)1237-43 | 72.88 (65.23 - 80.14) | Normal |
| AAA, EVAR | Surv_no_tx | Scott S, Batchelder A, Kirkbride D, et al. Eur J Vasc Endovasc Surg 2016;52(4):444-449 | 0.84 (0.83 - 0.85) | Triangle |
| AAA, EVAR | Surv_tx | Brewster D, Jones J, Chung T, et al. Ann Surg. 2006;244(3):426-38 | 0.88 (0.87 - 0.88) | Triangle |
| AAA, EVAR | QoL_no_tx | Experts | 0.76 (0.73 - 0.79) | Triangle |
| AAA, EVAR | QoL_tx | Experts | 0.89 (0.87 - 0.91) | Triangle |
| AAA, EVAR | Age | Scott S, Batchelder A, Kirkbride D, et al. Eur J Vasc Endovasc Surg 2016;52(4):444-449 | 72.85 (71.62 - 74.63) | Normal |
| Cox arthrosis, hip replacement | Surv_no_tx | Clinical insight | 1.00 (1.00 - 1.00) | Triangle |
| Cox arthrosis, hip replacement | Surv_tx | Clinical insight | 1.00 (1.00 - 1.00) | Triangle |
| Cox arthrosis, hip replacement | QoL_no_tx | Experts | 0.73 (0.70 - 0.76) | Triangle |
| Cox arthrosis, hip replacement | QoL_tx | Experts | 0.92 (0.90 - 0.94) | Triangle |
| Cox arthrosis, hip replacement | Age | Klop C, de Vries F, Lalmohamed A, et al. Calcif Tissue Int. 2012 Dec;91(6):387-94 | 69.53 (62.16 - 78.99) | Normal |
| Knee arthrosis, knee replacement | Surv_no_tx | Clinical insight | 1.00 (1.00 - 1.00) | Triangle |
| Knee arthrosis, knee replacement | Surv_tx | Clinical insight | 1.00 (1.00 - 1.00) | Triangle |
| Knee arthrosis, knee replacement | QoL_no_tx | Experts | 0.73 (0.70 - 0.75) | Triangle |
| Knee arthrosis, knee replacement | QoL_tx | Experts | 0.91 (0.90 - 0.93) | Triangle |
| Knee arthrosis, knee replacement | Age | DHD | 68.01 (67.88 - 68.09) | Normal |
| Benign prostatic hyperplasia, TURP | Surv_no_tx | Clinical insight | 1.00 (1.00 - 1.00) | Triangle |
| Benign prostatic hyperplasia, TURP | Surv_tx | Clinical insight | 1.00 (1.00 - 1.00) | Triangle |
| Benign prostatic hyperplasia, TURP | QoL_no_tx | Experts | 0.80 (0.77 - 0.83) | Triangle |
| Benign prostatic hyperplasia, TURP | QoL_tx | Experts | 0.91 (0.89 - 0.93) | Triangle |
| Benign prostatic hyperplasia, TURP | Age | DHD | 70.99 (70.86 - 71.17) | Normal |
| Benign uterus tumor, hysterectomy | Surv_no_tx | Clinical insight | 1.00 (1.00 - 1.00) | Triangle |
| Benign uterus tumor, hysterectomy | Surv_tx | Clinical insight | 1.00 (1.00 - 1.00) | Triangle |
| Benign uterus tumor, hysterectomy | QoL_no_tx | Experts | 0.78 (0.76 - 0.80) | Triangle |
| Benign uterus tumor, hysterectomy | QoL_tx | Experts | 0.91 (0.89 - 0.92) | Triangle |
| Benign uterus tumor, hysterectomy | Age | DHD | 50.98 (50.69 - 51.28) | Normal |
| Cervical cancer, resection | Surv_no_tx | Calculated from tx effect | 0.83 (0.79 - 0.87) | Calc_from_tx_eff |
| Cervical cancer, resection | Surv_tx | IKNL | 0.93 (0.92 - 0.93) | Triangle |
| Cervical cancer, resection | Tx_eff | Bansal N, Herzog T, Shaw R, et al. Am J Obstet Gynecol 2009;201:485.e1-9 | 0.41 (0.32 - 0.53) | Lognormal |
| Cervical cancer, resection | QoL_no_tx | Experts | 0.68 (0.64 - 0.71) | Triangle |
| Cervical cancer, resection | QoL_tx | Experts | 0.88 (0.86 - 0.90) | Triangle |
| Cervical cancer, resection | Age | Landoni F, Maneo A, Colombo A, et al. Lancet 1997;351(9077):535-40 | 51.76 (50.46 - 53.21) | Normal |
| Cervical cancer, resection | Time_noeff_Surv | Nanthamongkolkul K, Hanprasertpong J. J Gynecol Oncol. 2015;26(4):262-9 | 7.90 (5.27 - 11.00) | Triangle |
| NSCLC, lobectomy | Surv_no_tx | Ginsberg R, Rubinstein L. Ann Thorac Surg 1995;60(3):615-22 | 0.84 (0.80 - 0.88) | Triangle |
| NSCLC, lobectomy | Surv_tx | IKNL | 0.92 (0.91 - 0.92) | Triangle |
| NSCLC, lobectomy | QoL_no_tx | Experts | 0.54 (0.51 - 0.57) | Triangle |
| NSCLC, lobectomy | QoL_tx | Experts | 0.77 (0.75 - 0.80) | Triangle |
| NSCLC, lobectomy | Age | Kann B, Verma V, Stahl J, et al. Radiother Oncol 2019;134:44-49 | 78.99 (78.46 - 79.50) | Normal |
| NSCLC, lobectomy | Time_noeff_Surv | Salomaa E, Sällinen S, Hiekkanen H, et al. Chest 2005;128(4):2282-8 | 12.06 (7.83 - 16.58) | Triangle |
| Severe meniscusrupture, (partial) meniscectomy | Surv_no_tx | Clinical insight | 1.00 (1.00 - 1.00) | Triangle |
| Severe meniscusrupture, (partial) meniscectomy | Surv_tx | Clinical insight | 1.00 (1.00 - 1.00) | Triangle |
| Severe meniscusrupture, (partial) meniscectomy | QoL_no_tx | Experts | 0.83 (0.80 - 0.85) | Triangle |
| Severe meniscusrupture, (partial) meniscectomy | QoL_tx | Experts | 0.95 (0.93 - 0.96) | Triangle |
| Severe meniscusrupture, (partial) meniscectomy | Age | DHD | 42.01 (41.77 - 42.20) | Normal |
| HNP, discectomy | Surv_no_tx | Clinical insight | 1.00 (1.00 - 1.00) | Triangle |
| HNP, discectomy | Surv_tx | Clinical insight | 1.00 (1.00 - 1.00) | Triangle |
| HNP, discectomy | QoL_no_tx | Experts | 0.67 (0.64 - 0.71) | Triangle |
| HNP, discectomy | QoL_tx | Experts | 0.92 (0.90 - 0.93) | Triangle |
| HNP, discectomy | Age | DHD | 49.00 (48.61 - 49.40) | Normal |
| Thyroid adenoma, hemithyreoïdectomy | Surv_no_tx | Clinical insight | 1.00 (1.00 - 1.00) | Triangle |
| Thyroid adenoma, hemithyreoïdectomy | Surv_tx | Clinical insight | 1.00 (1.00 - 1.00) | Triangle |
| Thyroid adenoma, hemithyreoïdectomy | QoL_no_tx | Experts | 0.88 (0.86 - 0.91) | Triangle |
| Thyroid adenoma, hemithyreoïdectomy | QoL_tx | Experts | 0.95 (0.94 - 0.96) | Triangle |
| Thyroid adenoma, hemithyreoïdectomy | Age | Attaallah W, Erel S, Canturk N, et al. Neth J Med.2015 Jan;73(1):17-22 | 43.16 (41.66 - 44.28) | Normal |
| Thyroid cancer, resection | Surv_no_tx | Davies L, Welch H. Arch Otolaryngol Head Neck Surg. 2010;136(5):440-4 | 1.00 (0.99 - 1.00) | Triangle |
| Thyroid cancer, resection | Surv_tx | Davies L, Welch H. Arch Otolaryngol Head Neck Surg. 2010;136(5):440-4 | 1.00 (1.00 - 1.00) | Triangle |
| Thyroid cancer, resection | QoL_no_tx | Experts | 0.75 (0.71 - 0.78) | Triangle |
| Thyroid cancer, resection | QoL_tx | Experts | 0.90 (0.89 - 0.92) | Triangle |
| Thyroid cancer, resection | Age | Shin D, Cho J, Kim S, et al. Ann Surg Oncol. 2013;20(8):2468-76 | 46.51 (46.24 - 46.81) | Normal |
| Thyroid cancer, resection | Time_noeff_Surv | Shin D, Cho J, Kim S, et al. Ann Surg Oncol. 2013;20(8):2468-76 | 31.86 (16.65 - 46.63) | Triangle |
| Instable AP, PCI | Surv_no_tx | Calculated from tx effect | 0.92 (0.91 - 0.94) | Calc_from_tx_eff |
| Instable AP, PCI | Surv_tx | NHR | 0.95 (0.94 - 0.95) | Triangle |
| Instable AP, PCI | Tx_eff | Keeley E, Boura J, Grines C, et al. The Lancet 2003;361(9351):13-20 | 0.71 (0.57 - 0.82) | Lognormal |
| Instable AP, PCI | QoL_no_tx | Experts | 0.75 (0.72 - 0.78) | Triangle |
| Instable AP, PCI | QoL_tx | Experts | 0.89 (0.88 - 0.91) | Triangle |
| Instable AP, PCI | Age | NHR | 65.65 (52.90 - 74.94) | Normal |
| Instable AP, CABG | Surv_no_tx | Calculated from tx effect | 0.96 (0.94 - 0.97) | Calc_from_tx_eff |
| Instable AP, CABG | Surv_tx | NHR | 0.97 (0.97 - 0.98) | Triangle |
| Instable AP, CABG | Tx_eff | Yusuf S, Zucker D, Passamani E, et al. The Lancet 1994;344(8922):563-570 | 0.61 (0.49 - 0.76) | Lognormal |
| Instable AP, CABG | QoL_no_tx | Experts | 0.71 (0.67 - 0.75) | Triangle |
| Instable AP, CABG | QoL_tx | Experts | 0.86 (0.84 - 0.88) | Triangle |
| Instable AP, CABG | Age | NHR | 66.74 (59.23 - 77.05) | Normal |
| Pacemaker implantation | Surv_no_tx | Calculated from tx effect | 0.83 (0.73 - 0.89) | Calc_from_tx_eff |
| Pacemaker implantation | Surv_tx | Brunner M, Olschewski M, Geibel A, et al. Eur Heart J 2004;25(1):88-95 | 0.92 (0.92 - 0.92) | Triangle |
| Pacemaker implantation | Tx_eff | Moss A, Hall W, Cannom D, et al. N Engl J Med 1996;335(26)1933-40 | 0.46 (0.27 - 0.75) | Lognormal |
| Pacemaker implantation | QoL_no_tx | Experts | 0.73 (0.71 - 0.76) | Triangle |
| Pacemaker implantation | QoL_tx | Experts | 0.91 (0.90 - 0.93) | Triangle |
| Pacemaker implantation | Age | NHR | 75.98 (75.78 - 76.22) | Normal |
| PAD F2, bypass | Surv_no_tx | Calculated from tx effect | 0.90 (0.89 - 0.90) | Calc_from_tx_eff |
| PAD F2, bypass | Surv_tx | Muluk S, Muluk V, Kelley M, et al. J Vasc Surg 2001;33(2):251-7 | 0.90 (0.89 - 0.90) | Triangle |
| PAD F2, bypass | Tx_eff | Clinical insight | 1.00 (0.98 - 1.03) | Lognormal |
| PAD F2, bypass | QoL_no_tx | Experts | 0.62 (0.58 - 0.65) | Triangle |
| PAD F2, bypass | QoL_tx | Experts | 0.80 (0.78 - 0.82) | Triangle |
| PAD F2, bypass | Age | Muluk S, Muluk V, Kelley M, et al. J Vasc Surg 2001;33(2):251-7 | 64.71 (64.41 - 64.98) | Normal |
| Carotid endarterectomy | Surv_no_tx | European Carotid Surgery Trialists' Collaborative Group The Lancet 1998;351(9113):1379-1387 | 0.96 (0.96 - 0.97) | Triangle |
| Carotid endarterectomy | Surv_tx | European Carotid Surgery Trialists' Collaborative Group The Lancet 1998;351(9113):1379-1387 | 0.97 (0.97 - 0.97) | Triangle |
| Carotid endarterectomy | QoL_no_tx | Experts | 0.67 (0.64 - 0.70) | Triangle |
| Carotid endarterectomy | QoL_tx | Experts | 0.85 (0.84 - 0.87) | Triangle |
| Carotid endarterectomy | Age | European Carotid Surgery Trialists' Collaborative Group The Lancet 1998;351(9113):1379-1387 | 62.50 (62.22 - 62.86) | Normal |
| Carotid endarterectomy | Time_noeff_QoL | European Carotid Surgery Trialists' Collaborative Group The Lancet 1998;351(9113):1379-1387 | 60.97 (34.49 - 93.58) | Triangle |
| Breast cancer, resection | Surv_no_tx | Calculated from tx effect | 0.97 (0.96 - 0.98) | Calc_from_tx_eff |
| Breast cancer, resection | Surv_tx | IKNL | 0.98 (0.98 - 0.98) | Triangle |
| Breast cancer, resection | Tx_eff | Soran A, Ozmen V, Ozbas S, et al. Ann Surg Oncol 2018;25:3141-3149 | 0.66 (0.52 - 0.92) | Lognormal |
| Breast cancer, resection | QoL_no_tx | Experts | 0.69 (0.66 - 0.71) | Triangle |
| Breast cancer, resection | QoL_tx | Experts | 0.89 (0.87 - 0.90) | Triangle |
| Breast cancer, resection | Age | Bleicher R, Ruth K, Sigurdson E, et al. JAMA Onco. 2016;2(3):330-339 | 60.51 (60.22 - 60.96) | Normal |
| Breast cancer, resection | Time_noeff_Surv | Bleicher R, Ruth K, Sigurdson E, et al. JAMA Onco. 2016;2(3):330-339 | 21.43 (18.42 - 25.07) | Triangle |
| Breast cancer, mastectomy | Surv_no_tx | Calculated from tx effect | 0.97 (0.96 - 0.98) | Calc_from_tx_eff |
| Breast cancer, mastectomy | Surv_tx | IKNL | 0.98 (0.98 - 0.98) | Triangle |
| Breast cancer, mastectomy | Tx_eff | Soran A, Ozmen V, Ozbas S, et al. Ann Surg Oncol 2018;25:3141-3149 | 0.65 (0.49 - 0.81) | Lognormal |
| Breast cancer, mastectomy | QoL_no_tx | Experts | 0.65 (0.62 - 0.68) | Triangle |
| Breast cancer, mastectomy | QoL_tx | Experts | 0.81 (0.80 - 0.83) | Triangle |
| Breast cancer, mastectomy | Age | Bleicher R, Ruth K, Sigurdson E, et al. JAMA Onco. 2016;2(3):330-339 | 60.55 (60.10 - 60.94) | Normal |
| Breast cancer, mastectomy | Time_noeff_Surv | Bleicher R, Ruth K, Sigurdson E, et al. JAMA Onco. 2016;2(3):330-339 | 21.81 (18.08 - 24.42) | Triangle |
| Varicose veins, stripping | Surv_no_tx | Clinical insight | 1.00 (1.00 - 1.00) | Triangle |
| Varicose veins, stripping | Surv_tx | Clinical insight | 1.00 (1.00 - 1.00) | Triangle |
| Varicose veins, stripping | QoL_no_tx | Experts | 0.87 (0.86 - 0.89) | Triangle |
| Varicose veins, stripping | QoL_tx | Experts | 0.95 (0.93 - 0.96) | Triangle |
| Varicose veins, stripping | Age | Oriol Parés J, Juan J, Tellez R, et al. Ann Surg. 2010 Apr;251(4):624-31 | 50.13 (48.49 - 51.96) | Normal |
| Colon carcinoma, colectomy without stoma | Surv_no_tx | Sud A, Jones M, Broggio J, et al. Ann. Oncol. 2020;31(8):1065-1074 | 0.94 (0.91 - 0.97) | Normal |
| Colon carcinoma, colectomy without stoma | Surv_tx | IKNL | 0.96 (0.96 - 0.96) | Normal |
| Colon carcinoma, colectomy without stoma | QoL_no_tx | Experts | 0.66 (0.62 - 0.69) | Triangle |
| Colon carcinoma, colectomy without stoma | QoL_tx | Experts | 0.89 (0.87 - 0.91) | Triangle |
| Colon carcinoma, colectomy without stoma | Age | Verwaal V, Bruin S, Boot H, et al. Ann Surg Oncol. 2008;15(9):2426-32 | 53.77 (51.36 - 56.82) | Normal |
| Colon carcinoma, colectomy without stoma | Time_noeff_Surv | Hangaard Hansen C, Gögenur M, Rvilling Madsen M, et al. Eur J Surg Oncol 2018;44(10):1479-1485 | 24.01 (11.61 - 44.02) | Triangle |
| Colon carcinoma, colectomy with stoma | Surv_no_tx | Sud A, Jones M, Broggio J, et al. Ann. Oncol. 2020;31(8):1065-1074 | 0.94 (0.91 - 0.97) | Normal |
| Colon carcinoma, colectomy with stoma | Surv_tx | IKNL | 0.96 (0.96 - 0.96) | Normal |
| Colon carcinoma, colectomy with stoma | QoL_no_tx | Experts | 0.65 (0.61 - 0.68) | Triangle |
| Colon carcinoma, colectomy with stoma | QoL_tx | Experts | 0.79 (0.77 - 0.81) | Triangle |
| Colon carcinoma, colectomy with stoma | Age | Verwaal V, Bruin S, Boot H, et al. Ann Surg Oncol. 2008;15(9):2426-32 | 53.90 (51.34 - 56.84) | Normal |
| Colon carcinoma, colectomy with stoma | Time_noeff_Surv | Hangaard Hansen C, Gögenur M, Rvilling Madsen M, et al. Eur J Surg Oncol 2018;44(10):1479-1485 | 21.42 (10.97 - 43.29) | Triangle |
| Prostate carcinoma, prostatectomy | Surv_no_tx | Calculated from tx effect | 0.96 (0.92 - 0.98) | Calc_from_tx_eff |
| Prostate carcinoma, prostatectomy | Surv_tx | IKNL | 0.96 (0.94 - 0.97) | Triangle |
| Prostate carcinoma, prostatectomy | Tx_eff | Fossati N, Sofia Rossi M, Cucchiara V, et al. Urol Oncol. 2017;35:150.39-150.e15 | 0.95 (0.48 - 1.63) | Triangle |
| Prostate carcinoma, prostatectomy | QoL_no_tx | Experts | 0.76 (0.74 - 0.79) | Triangle |
| Prostate carcinoma, prostatectomy | QoL_tx | Experts | 0.84 (0.82 - 0.86) | Triangle |
| Prostate carcinoma, prostatectomy | Age | DHD | 71.00 (70.84 - 71.17) | Normal |
| Prostate carcinoma, prostatectomy | Time_noeff_Surv | Fossati N, Sofia Rossi M, Cucchiara V, et al. Urol Oncol. 2017;35:150.39-150.e15 | 50.76 (50.22 - 50.99) | Triangle |

**Parameter** **abbreviations**: Surv_no_tx: The expected survival of the patients before the surgery; Surv_tx: The expected survival of the patients after the surgery; Tx_eff: The treatment effect of the surgery; QoL_no_tx: The quality of life of the patients before the surgery; QoL_tx: The quality of life of the patients after the surgery; Age: Average age of the patient population; Time_noeff_Surv: The time until no effect on the survival is expected anymore by the surgery; Time_noeff_QoL:The time until no effect on the quality of life is expected by surgery.
