# Supplementary material for: Minimising population health loss in times of scarce surgical capacity: a modelling study for surgical procedures performed in nonacademic hospitals
Source: BMC Health Serv Res. 2022 Nov 30;22:1456. doi: 10.1186/s12913-022-08854-x (PMC9713162; doi:10.1186/s12913-022-08854-x)

Additional file 3

Panel members and the calibrated visual analogue scale.

Value Based Operation Room Triage team collaborators: the panel of physicians who participated in the Welphi rounds to estimate the QoL weights:

Arend Arends, geriatrician

Brigitte Haberkorn, medical oncologist

Charles van Rossem, surgeon

Gabrielle H. van Ramshorst, gastrointestinal and oncological surgeon

Han de Graaff, anaesthesiologist

Harm Sleeboom, medical oncologist

Jonne Postema, anaesthesiologist

Josien Terwisscha van Scheltinga, gynaecologist

Linda Valk-Kleibreuker, rehabilitation specialist

Marco Hoedt, surgeon

Martin Baartmans, paediatrician

Mike Nieboer, orthopaedic surgeon

Miriam Faes, geriatrician

Nieke Oversier, anaesthesiologist

Niels Schep, traumasurgeon

Onno Schuitema, plastic surgeon

Patricia MacLean, rheumatologist

Patrick Schouwenberg, intensivist

Paul ten Koppel, otorhinolaryngologist

Pieter Bakx, orthopaedic surgeon

Sjaak Pouwels, resident not in training Intensive Care

Suze Raaff, general practitioner

Taco Nieboer, general practitioner

Tietse van dorp, radiologist

Willem Maarten Bosman, surgeon

Calibrated visual analogue scale for the collection of QoL data:


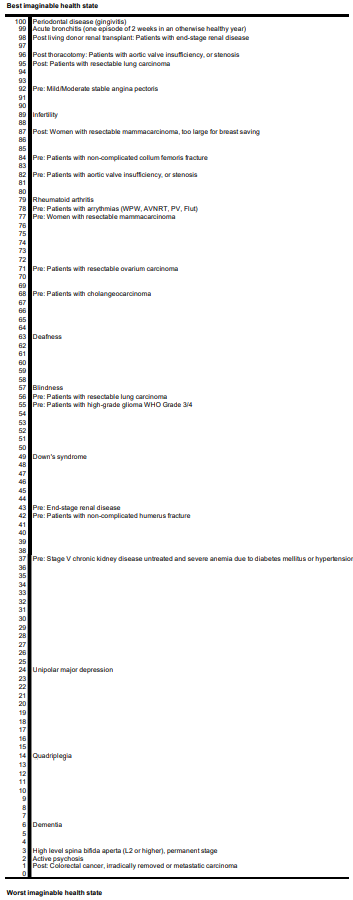

Supplement: Supplementary file 3 — Additional file 3. [file 12913_2022_8854_MOESM3_ESM.docx]
