# Supplementary material for: Minimising population health loss in times of scarce surgical capacity: a modelling study for surgical procedures performed in nonacademic hospitals
Source: BMC Health Serv Res. 2022 Nov 30;22:1456. doi: 10.1186/s12913-022-08854-x (PMC9713162; doi:10.1186/s12913-022-08854-x)
Supplement: Supplementary file 4 — Additional file 4. [file 12913_2022_8854_MOESM4_ESM.docx]

Additional file 4

Evaluation of the agreement on QoL estimates between two expert panels.


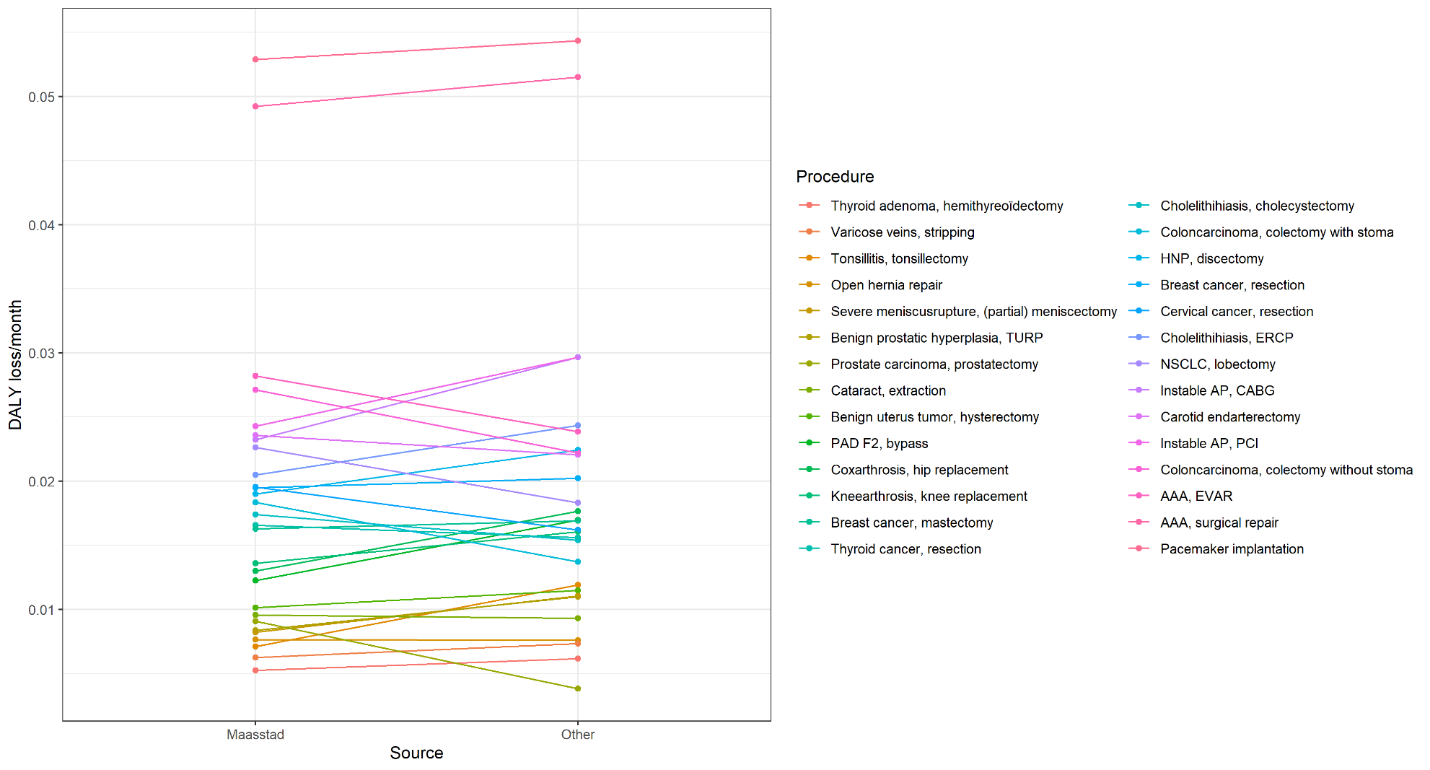


Figure A4.1. The difference in DALY loss/month, which we use as a measure of urgency (y-axis), is shown for the n = 28 nonacademic surgical procedures between the ‘Maasstad experts’ and the ‘Other experts’ (x-axis). See Table 1 from the manuscript for the abbreviations used.


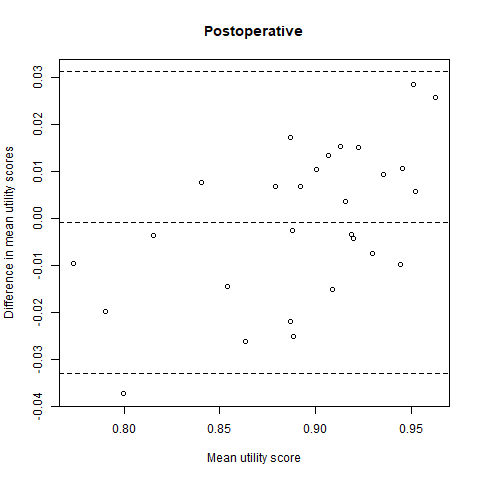

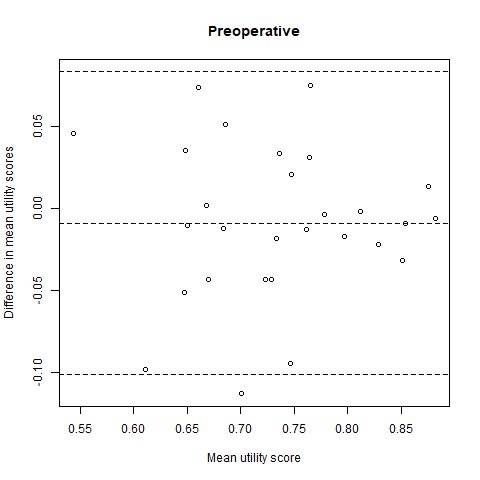


Figure A4.2. The Bland-Altman plot of the quality of life estimates from the ‘Maasstad experts’ and ‘Other experts’, stratified for the preoperative (left) and postoperative (right) state. On the y-axis the overall mean difference in quality of life estimates is shown and the x-axis represents the mean quality of life estimates based on data from both panels. The dashed horizontal lines represent the Bland-Altman bias and 95% limits of agreement.

The overall mean difference for the preoperative state is -0.01 (95% CI -0.10–0.08) and for the postoperative state -0.00 (95% CI -0.03–0.03). The overall mean difference of quality of life estimates between the two panels was 0.00 (95% CI -0.01-0.00). From this we conclude that the QoL estimates between both panels were highly comparable.
